# High Expression of KCa3.1 in Patients with Clear Cell Renal Carcinoma Predicts High Metastatic Risk and Poor Survival

**DOI:** 10.1371/journal.pone.0122992

**Published:** 2015-04-07

**Authors:** Maj Rabjerg, Aida Oliván-Viguera, Lars Koch Hansen, Line Jensen, Linda Sevelsted-Møller, Steen Walter, Boye L. Jensen, Niels Marcussen, Ralf Köhler

**Affiliations:** 1 Department of Pathology, Odense University Hospital, DK-5000 Odense C, Denmark; 2 Aragon Institute of Health Sciences I+CS/IIS, 50009 Zaragoza, Spain; 3 Department of Cardiovascular and Renal Research, Institute of Molecular Medicine, University of Southern Denmark, DK-5000 Odense C, Denmark; 4 Department of Urology, Odense University Hospital, DK-5000 Odense C, Denmark; 5 Fundación Agencia Aragonesa para la Investigación y Desarrollo (ARAID), 50009 Zaragoza, Spain; University of Miami School of Medicine, UNITED STATES

## Abstract

**Background:**

Ca^2+^-activated K^+^ channels have been implicated in cancer cell growth, metastasis, and tumor angiogenesis. Here we hypothesized that high mRNA and protein expression of the intermediate-conductance Ca^2+^-activated K^+^ channel, KCa3.1, is a molecular marker of clear cell Renal Cell Carcinoma (ccRCC) and metastatic potential and survival.

**Methodology/Principal Findings:**

We analyzed channel expression by qRT-PCR, immunohistochemistry, and patch-clamp in ccRCC and benign oncocytoma specimens, in primary ccRCC and oncocytoma cell lines, as well as in two ccRCC cell lines (Caki-1 and Caki-2). CcRCC specimens contained 12-fold higher mRNA levels of KCa3.1 than oncocytoma specimens. The large-conductance channel, KCa1.1, was 3-fold more highly expressed in ccRCC than in oncocytoma. KCa3.1 mRNA expression in ccRCC was 2-fold higher than in the healthy cortex of the same kidney. Disease specific survival trended towards reduction in the subgroup of high-KCa3.1-expressing tumors (p<0.08 vs. low-KCa3.1-expressing tumors). Progression-free survival (time to metastasis/recurrence) was reduced significantly in the subgroup of high-KCa3.1-expressing tumors (p<0.02, vs. low-KCa3.1-expressing tumors). Immunohistochemistry revealed high protein expression of KCa3.1 in tumor vessels of ccRCC and oncocytoma and in a subset of ccRCC cells. Oncocytoma cells were devoid of KCa3.1 protein. In a primary ccRCC cell line and Caki-1/2-ccRCC cells, we found KCa3.1-protein as well as TRAM-34-sensitive KCa3.1-currents in a subset of cells. Furthermore, Caki-1/2-ccRCC cells displayed functional Paxilline-sensitive KCa1.1 currents. Neither KCa3.1 nor KCa1.1 were found in a primary oncocytoma cell line. Yet KCa-blockers, like TRAM-34 (KCa3.1) and Paxilline (KCa1.1), had no appreciable effects on Caki-1 proliferation in-vitro.

**Conclusions/Significance:**

Our study demonstrated expression of KCa3.1 in ccRCC but not in benign oncocytoma. Moreover, high KCa3.1-mRNA expression levels were indicative of low disease specific survival of ccRCC patients, short progression-free survival, and a high metastatic potential. Therefore, KCa3.1 is of prognostic value in ccRCC.

## Introduction

Clear cell Renal Cell Carcinoma (ccRCC) is the most common malignant tumor of the adult kidney [1]. Patients with ccRCC respond poorly to chemotherapy or radiotherapy and overall survival is highly variable ranging from 1–10 years. Moreover, disease progression in the individual patients is uncertain because of a similarly variable risk of developing metastasis. Molecular predictors of disease progression and metastasis may be of value to adjust therapies in the individual patient and predict survival and outcome. Therefore, we set up a study to identify new molecular markers of disease progression and ccRCC-specific molecular mechanisms that may provide new targeted treatment options. One candidate is the intermediate-conductance calcium/calmodulin-activated potassium channel, KCa3.1, encoded by the KCNN4 gene [2,3]. KCa3.1 is expressed in red and white blood cell lineages and in epithelia of secretory organs, such as the salivary gland, mammary gland, trachea, and prostate, as well as in the intestinal crypts and the vascular endothelium [4,5]. The tubular system of the kidney is believed to be devoid of KCa3.1 channels [5] while some channel expression is present in renal vasculature. KCa3.1 channels have been reported to be up-regulated in disease states characterized by abnormal cell proliferations such as neointima formation [6,7] and organ fibrosis [8], and, important for the present study, in several solid cancers; prostate, hepatocellular carcinoma, endometrial, mammary carcinoma and glioblastoma [9–14], several cancer cell lines [15–19], tumor vessels, proliferating endothelial cells [20,21], and activated T cells [22–25]. An established cellular mechanism underlying this up-regulation of KCa3.1-mRNA is activation of the mitogen-activated protein (MAP) kinase signaling and resultant AP-1-mediated mRNA transcription [4,6,26]. At the cell physiological level, KCa3.1 channels provide K^+^ efflux and hyperpolarization after activation by the release of Ca^2+^ from intracellular stores, thus regulating e.g. anion and water secretion in the gut [27], endothelium-derived hyperpolarization-mediated vasodilation [28], cell volume [29], and Ca^2+^ dynamics by providing a positive feedback as a cell membrane hyperpolarizing, countercurrent-producing channel [30,31]. With respect to cell proliferation and migration, several studies have suggested KCa3.1-functions to be required for Ca^2+^-sensitive steps of cell cycle progression, since a hyperpolarized state due to K^+^ channel activation enhances calcium entry and thereby calcium homeostasis, which is critical in controlling the passage of cells through G0/G1 or the G1/S phase transition [32,33]. Moreover, KCa3.1 as a cell volume-regulating channel could influence cell volume adjustment during mitosis as well as migration [12,34]. Pharmacological in-vitro studies show that inhibition of KCa3.1 channel activity suppresses proliferation of pancreatic cancer [17] and hepatocellular carcinoma cells [11], reduces cell motility in glioblastoma [10], and also reduces endothelial cell proliferation and neo-angiogenesis on matrigels [20]. Thus, KCa3.1 emerges as a potential molecular marker of tumor growth and progression as well as a potential treatment target as supported by recent studies on glioblastoma showing that TRAM-34 reduces tumor cell invasiveness [12,34]. Interestingly, blockade of KCa3.1 in a subtype of human natural killer cell has been shown to increase the killing of tumor cells [35].

Another candidate is KCa1.1, the large-conductance, voltage and Ca^+^-activated potassium channel (also known as “big K^+^ conductance” channel, BK, or “Maxi-K”), whose K^+^ conducting alpha-subunit is encoded by the KCNMA1-gene [36,37]. The channel is expressed in many different cell types and is known to modulate numerous physiological processes, for instance, the smooth muscle tone of the arteries and synaptic neurotransmitter release [38–41]. Importantly and in contrast to KCa3.1, the KCa1.1 channel is expressed in several segments of the tubular system of the kidney, particularly at the apical membrane of the intercalated cells of the aldosterone-sensitive distal nephron and collecting duct, where it contributes to the excretion of potassium[42,43]. Besides these physiological functions, there is growing evidence that KCa1.1 channels are associated with altered cell cycle progression, cell proliferation and oncogenesis. [44,45]. Indeed, KCa1.1 channel activation has been shown to drive tumor cell proliferation in astrocytoma [46], prostate cancer [47,48] and breast cancer [49].

The roles of KCa3.1 and KCa1.1 in renal cancers and their significance for disease progression, metastasis, and survival have not been studied yet. In the present study we test the hypothesis that these KCa channels, in particularly KCa3.1, serve as diagnostic and prognostic markers of ccRCC.

## Materials and Methods

### Tumor and renal cortex samples and collection of patient data

Paired frozen tissue from tumor and cortex diagnosed with either ccRCC or oncocytoma collected in the years 2001–2012 were included in the study. For inclusion, tissue from both tumor and non-tumorous renal cortex had to be available together with clinical data and follow-up information. Patients treated preoperatively with adjuvant therapy were excluded. In total, we included 97 patients with ccRCC and 11 patients with oncocytoma. All cases were available as formalin-fixed paraffin-embedded tissue and all slides were evaluated for histology and Fuhrman grading by two pathologists (N.M. and M.R.). TNM stages (**T**umor size/no. affected lymph **N**odes/distant **M**etastasis) were updated to follow the latest guidelines (7^th^ Edition 2009). See Table 1 for demographic patient data.

**Table 1:** Clinical and demographic data of ccRCC and oncocytoma.

Clinical and demographic data	ccRCC (n = 97)		Oncocytoma (n = 11)	
	n (%)	Mean; median (range)	n (%)	Mean; median (range)
**Sex**				
Male	43 (44)		5 (45)	
Female	54 (56)		6 (55)	
**Age, yrs**		62;64(28–86)		65;67(39–81)
≤ 64	49 (51)		5 (45)	
> 64	48 (49)		6 (55)	
**Symptom presentation**				
Yes	47 (48)		2 (18)	
No (incidental findings)	50 (52)		9 (82)	
**Tumor size, cm**		7,22; 6,5 (2–22)		5,88;4 (2,5–15)
< 7	54 (56)		8 (73)	
≥ 7	42 (43)		3 (27)	
**TNM stage**				
I	40 (41)			
II	18 (19)			
III	31 (32)			
IV	8 (8)			
**Fuhrman grade**				
G1+G2	53 (55)			
G3+G4	44 (45)			
**Vessel invasion**				
Yes	21 (22)			
No	76 (78)			
**Adjuvant therapy**				
Yes	24 (25)			
No	73 (75)			
**Follow-up (months)**				
		33; 29(0.92–106)	21;4 (1.2–77.5)	
**Progression Free Survival (months)**				
		28;25 (0.03–86)		
**Death**				
	34 (35)		2 (18)	
**Death from RCC**				
	23 (24)		0 (0)	

### Ethics statement

Human tissue collection was performed by the Department of Pathology, Odense University Hospital. All patients gave informed and written consent and the study was approved by the local ethics committee (notification number 29573, Region of Southern Denmark) and the local data protection agency (file number 13/14405, Odense University Hospital).

### Cell cultures

Two commercially available ccRCC lines (Caki-1 and Caki-2, American Type Culture Collection (ATCC), Rockville, MD) were cultured in a 1:1 mixture of DMEM (Dulbecco’s modified Eagle medium) containing 25 mM HEPES (4-(2-hydroxyethyl)-1-piperazineethanesulfonic acid) and DMEM+ GlutaMAX™ (Life Technologies), supplemented with 10% newborn calf serum and 1% penicillin/streptomycin. Cells were cultured continuously in a humidified 5% CO_2_ incubator at 37°C.

One primary ccRCC cell line and one oncocytoma cell line were prepared from surgical specimens. Briefly, a sample of fresh tumor tissue was sliced in a petri dish with scalpels. After washing in phosphate-buffered saline (PBS), the sample was incubated with collagenase type II (22 mg, 217 U/mg, Worthington Biochemical Corporation) dissolved in 8 ml HBSS (Hanks Balanced Salt Solution, Life Technologies) in a 37°C water bath for 30–40 min. After spinning, the supernatant was removed and the pellet was re-suspended with 15 ml of HBSS, filtered twice with a 100 μm and a 40 μm sieve to remove debris, centrifuged again, re-suspended with culture media and cultured as described above.

The presence of tumor cells was verified by immunocytochemistry of the markers PAX-8, CK7 and vimentin. ccRCC and oncocytoma primary cell lines were positive for all three markers, although most oncocytoma showed a more focal vimentin immunoreactivity [50] (data not shown). We did not observe CK7-stain in Caki1/2 cells (data not shown). In addition, electron microscopy on a cell pellet from the oncocytoma was performed to ensure the presence of mitochondria in the cytosol.

For immunocytochemistry and patch-clamp, cells were seeded onto coverslips and cultured until semi-confluence was reached or were used the same day for patch-clamp experiments on single cells.

### Reverse transcription and qRT-PCR

For qRT-PCR (quantitative reverse-transcription polymerase-chain-reaction), we isolated mRNA using the TRIZOL reagent (Invitrogen, United Kingdom). Concentrations of mRNA were measured in triplicate using a NanoDrop ND-1000 Spectrophotometer (NanoDrop Technologies, Inc., Wilmington, DE). Only isolated mRNA having a 260/280 nm purity ratio > 1.6 was used for complementary cDNA synthesis. First strand cDNA synthesis using iScript cDNA Synthesis Kit (Bio-Rad, CA USA) was carried out using 2000 ng of extracted mRNA from each case, following the manufacturer’s instructions. Thermal cycling conditions were as follows: 25°C for 5 minutes, 42°C for 30 minutes, and 85°C for 5 minutes.

qRT-PCR for KCa3.1 was carried out using an ABI PRISM 7900HT Sequence detecting system (Applied Biosystems, Foster City, CA, USA), 384 well TaqMan Custom Arrays (Applied Biosystems, Foster City, CA, USA), oligonucleotide primers and FAM labeled TaqMan probes (Table 2). Each of the 8 wells on every plate were loaded with 50 μl of cDNA mixed with 50 μl TaqMan Universal PCR Master Mix 2x (Applied Biosystems) according to the manufacturer’s instructions, giving a total of 8 different samples on each qRT-PCR run. Tumor and cortex samples from the same patient were always run on the same plate. All measurements were done in duplicate. Thermal cycling conditions were as follows: 2 minutes at 50°C, 10 minutes at 94.5°C, followed by 50 cycles of 30 sec at 97°C, and 1 min at 60°C. RNase/DNase-free water was run as a non-template control together with samples synthesized without reverse transcriptase.

**Table 2:** Gene assays used for TaqMan RT-PCR.

**Gene symbol**	**Gene Name**	**Assay ID**	**NCBI Gene Reference Sequence**	**Function of Gene**	**Assay location (amplicon size)**
**KCNN4**	Intermediate calcium-activated potassium channel	Hs00158470_m1	NM_002250.2	Ion-channel	1218(148)
**HMBS**	Hydroxymethylbilane synthase	Hs00609297_m1	NM_000190.3 NM_001258208.1	Enzyme of heme biosynthetic pathway	186 (64) 186(64)
**TBP**	TATA box binding protein	Hs00427621_m1	NM_001172085.1 NM_003194.4	Modulates DNA binding activity	666(65) 868(65)
**PPIA**	Peptidylprolyl isomerase A	Hs99999904_m1	NM_021130.3	Cyclosporin binding-protein	433(98)

All PCR data were collected and evaluated with SDS 2.4 software (Applied Biosystems) and qBasePlus software (Biogazelle, Zwijnaarde, Belgium)[51]. The threshold was set manually to 0.3 to cut off background fluorescence. Ct values of < 40 were considered valid. Replicates were excluded if ΔCT between replicates was > 1 at Ct values of < 30 cycles and if ΔCT between replicates was > 1.5 at Ct values between 30 and 33 cycles. All replicates with Ct values above 33 cycles were considered valid. Values from the duplicate measurements were averaged. The gene expression stability (M) value for each gene was calculated using GeNorm software [52,53]. The ranking of the potential reference genes examined according to stability was as follows: TBP/PPIA > HMBS > LGALS3 > SPP1 > GAPDH, with GAPDH the least stably expressed gene[54]. The three reference genes estimated by the GeNorm software to provide the most reliable normalization factor were TBP (TATA box binding protein), PPIA (peptidylprolyl isomerase A), and HMBS (hydroxymethylbilane synthase). Normalization was done using the qBasePlus software according to the modified ΔΔCT method [55,56], where multiple reference genes are taken into account.

qRT-PCR for KCa1.1 was carried out using SYBR-green methodology in a 96-well format. All reagents were from Life Science, Bio-Rad Laboratories. We investigated a total of 42 cDNA samples from 10 ccRCC, and 11 oncocytoma patients together with corresponding normal renal cortex from the same patients. RNase/DNase-free water served as the non-template control and omission of RT served as—RT control on each 96 well plate. qRT-PCRs were run in triplicate on the Stratagene MX3000P qPCR instrument. The primers used for amplification of KCNMA1 were 5’ CATTTGGTGGAGAATTCAGG 3’ and 5’ GATGAAGAAGACCATGAAGAG 3’. Primers used for amplification of the reference gene, TBP, were 5’ CGGCTGTTTAACTTCGCTTC 3’ and 5’ CCAGCACACTCTTCTCAGCA 3’. Efficiencies of the primers were all between 90–100% (data not shown). Of note, we did not find significant differences in expression levels of TBP between the two tumor subtypes (p = 0.28).

Raw data were extracted using threshold detection at 4000. Cutoff values for including replicates were mean CT ± 2SD. Data were excluded when amplification slopes were not exponential and melting curves showed products of different sizes.

### Construction of Tissue Micro Arrays

Tissue micro arrays were constructed as follows: HE-stained slides from each tumor were evaluated and three representative tumor areas were encircled. Corresponding areas on the paraffin blocks were cored with a 0.6 mm puncher and placed together in new paraffin blocks. Furthermore, one single core from corresponding normal kidney tissue was cored and placed in each of the paraffin blocks.

### Immunohistochemistry and immunofluorescence

Tissue micro arrays were used for immunohistochemical staining of KCa3.1 and CD31 proteins, while slides with individual samples were used for immunofluorescence staining of the CD8 protein. Slides with individual samples were furthermore used for immunohistochemical staining of KCa1.1. We used the following antibodies: KCa3.1 (#AV35098 and #HPA053841, Sigma-Aldrich, Denmark), KCa1.1 (#P4872, Sigma-Aldrich, Denmark). CD8 (clone CD8/144B, mouse monoclonal, DAKO, Denmark) and CD31 (clone JC70A-Endothelial Cell, mouse monoclonal, DAKO, Denmark). The following antibody dilutions were used for immunohistochemistry: KCa3.1: #AV35098, 1: 2000 and #HPA053841, 1:500; KCa1.1: #P4872, 1:2000, CD31-mAB, 1:50. For immunofluorescence: #HPA053841, 1:125; CD8-mAB, 1:25; CD31-mAB, 1:12.5.

Immunohistochemistry was performed using the DAKO Powervision kit (DAKO, Denmark). Briefly, sections were driven through xylene and a gradient of ethanol to water and treated with 1.5% H_2_O_2_ for 10 min. Epitope retrieval was carried out with HIER (heat induced epitope retrieval) in T-EG buffer (Tris-EDTA-Glucose/Dextrose buffer, Fagron Nordic A/S, Denmark). The sections were then incubated with the primary antibody for 60 min. After 3x2 min wash in TNT buffer (Tris-NaCl-Tween buffer, Fagron Nordic A/S, Denmark), slides were treated with “Ready-to-use” Post-Blocking (Novocastra PowerVision+Poly-HRP IHC Detection Systems, Leica Biosystems, Germany) for 20 min, washed again and incubated with the secondary antibody Poly-HRP anti-mouse/rabbit IgG (Novocastra PowerVision+Poly-HRP IHC Detection Systems, Leica Biosystems) for 30 min. Sections were counterstained with hematoxylin and incubated with DAB+ for 10 minutes (DAB+ chromogen, DAKO Denmark A/S). Slides were then mounted with Aquatex (Merck, Darmstadt, Germany).

IHC on multi-block with different human tissues, in which KCa3.1 and KCa1.1 expression is established or absent, served as additional control experiments and the results were in line with IHC on these channels as shown in “The Swedish Human Protein Atlas” [56]. Please see S1 Fig for staining of KCa3.1 in human parotid gland and of KCa1.1 in the human renal tubular system. The antibodies against KCa3.1 used here are currently considered the most specific and feasible Abs available and were found useful to detect KCa3.1 in glioblastoma multiforme as reported previously by the group [57].

Immunofluorescence staining was done similarly on 7 ccRCC patients and 7 patients with oncocytoma. But instead of treating the sections with H_2_O_2_, sections were incubated in 2% bovine serum albumin after the HIER treatment. Fluorochrome-labeled antibodies (Alexa 488 and Alexa 594, Invitrogen, Eugene, Oregon, USA) were used as secondary antibodies (dilution 1:200). Nuclei were stained with DAPI (4’-6-Diamidino-2-phenylindole dihydrochloride, Vectashields, Vector Laboratories Inc., Burlingame, CA 94010). We used a Visiopharm integrated microscope and software module (Visiopharm, Hørsholm, Denmark) to measure fluorescent signals. 6–10 high power field images of each slide were obtained at the same intensity and fluorescent images were then analyzed with the CellProfiler Software (Broad Institute, Boston, MA, USA[58]) for determination of fluorescence intensities and quantification of co-localization of the fluorescent signal.

### Immunocytochemistry on primary ccRCC and oncocytoma cell lines

Confluent cells on coverslips were washed 1x in PBS and fixed in NBF (Neutral buffered formalin) for 5 min at room temperature. Cells were then washed in TBS (Tris buffer, Fagron Nordic A/S) for 2 min, followed by TNT (Tris-NaCl-Tween buffer, Fagron Nordic A/S) for 2 min and incubated with primary antibody (KCa3.1-AB, #AV35098, 1:2000, KCa1.1-AB #P4872, 1:1000) at room temperature for 60 min. Thereafter, sections were processed further using secondary antibodies as described above. Human embryonic kidney cells (HEK-293) stably expressing hKCa3.1 [59] served as a positive control for KCa3.1, whereas a glioblastoma cell line (U251 MG) served as a positive control for KCa1.1 [60]. Positive tumor cells were counted for each cell line in original magnification, 200x.

### Determination of microvessel density

Determination of microvessel density (MVD) was performed on the multi-blocks. Following staining with the CD31 antibody, CD31 positive vessels were counted in 10–15 randomly chosen samples of the whole core corresponding to 10–25% of the core area. For each tumor sample at least two evaluable cores were analyzed. To determine microvessel density, results were averaged and the number of CD31-positive vessels was divided by the total area of the samples of each core.

### Electrophysiology

Patch-clamp electrophysiology was performed as described previously [59]. In brief, whole-cell currents were recorded using either an EPC-10-USB amplifier (HEKA, Lambrecht-Pfalz, Germany) or an Axopatch patch-clamp amplifier (Axon Instruments, Foster City, CA, USA). Data were analyzed with either the Patchmaster software or the Clampfit 9.2 software. We used an intracellular KCl-pipette solution composed of (in mM): 140 KCl, 1 MgCl_2_, 2 EGTA, 1.71 CaCl_2_, and 5 HEPES (adjusted to pH 7.2 with KOH). The NaCl bath solution was composed of (mM): 140 NaCl, 5 KCl, 1 MgSO_4_, 1 CaCl_2_, 10 glucose and 10 HEPES (adjusted to pH 7.4 with NaOH). To ensure complete activation of KCa3.1 in some experimental series and to compare maximal current amplitudes in ccRCC, oncocytoma, and Caki cells, we performed the experiments in the presence of the KCa3.1-activator, SKA-31 (1 μM). We performed blocking experiments using the KCa3.1-blocker, TRAM-34 (1 μM) or the KCa1.1-blocker, Paxilline (1 μM). For such pharmacological manipulation, appropriate amounts of 1 mM stock solutions (1:10 pre-diluted with PBS) were added to the bath solution to give a final concentration of 1 μM. Electrophysiological properties of K^+^ currents in Caki-1 and Caki-2 cells were comparable and data were pooled. Oncocytoma cells were smaller than ccRCC cells as they had a substantially lower membrane capacitance (12±2 pF (n = 12) vs. 37±4 pF (n = 27); p<0.01).

### Cell proliferation assay

Cell proliferation was spectrophotometrically assessed as described previously [59]. Briefly, Caki-1 cells (500 cells/well) were seeded in 96-well plates and cultured with McCoy’s 5A with L-glutamine supplemented with 10% fetal bovine serum and 1% penicillin/streptomycin, in the presence of vehicle (DMSO, 0.1%), TRAM-34 (1 μM), Paxilline (10 μM), or a combination. Final DMSO concentrations were the same for all conditions. Non-heat inactivated calf serum served as standard mitogenic stimulus. Cells were fixed with formalin (10% in phosphate-buffered saline) at days 0,1,2,3, and 4, and stained for 5 min with 0.3% Janus B Green dye at room temperature with constant stirring. Cells were then de-stained with water. Dye was eluted with 200 μl/well of 0.5 M HCl for 15 min and absorbance at 595 nm was determined using a microplate reader (Sinergy HT, Biotek, USA). Absorbance values lower than control indicated less cell proliferation.

### Scratch assay

Cells from Caki-1 were seeded at the same density in 4 chambers (iBidi μ-Slide 8 well). At confluence, a scratch was made with a 10 μl pipette tip. Thereafter, chambers were washed with PBS to remove detached cells. We added either vehicle (DMSO), Paxilline (1 μM), RA-2 (1 μM) or a combination of both blockers. Time-lapse recordings using a Nikon TE 2000E microscope and LAS AF V2.3.5 software (Leica Microsystems CMS GmbH) were done at 5x magnification and 6 frames per hour. The degree of wound closing (% of remaining cell-free area) was measured after 0, 12, 24, 30, 36 and 48 hours using the ImageJ software. The experiment was repeated twice.

### Drugs and Kits

TRAM-34 and SKA-31 were kind gifts from Dr. Heike Wulff, Department of Pharmacology, University of California Davis, California, USA. Paxilline was purchased from Tocris Bioscience (Bristol, United Kingdom). TRIZOL reagent was purchased from Invitrogen (United Kingdom). The RNase-Free DNase Set (Qiagen, Germany) was used for DNase digestion. Complementary DNA (cDNA) was synthesized using iScript cDNA Synthesis Kit (Bio-Rad, CA, USA). The new negative-gating modulator of KCa3.1, RA-2 (1,3-phenylenebis(methylene) bis(3-fluoro-4-hydroxybenzoate), was synthesized in house [61]

### Statistics

Data are represented as mean ± SEM. For comparison of two data sets with even or uneven variance, we used the Wilcoxon-signed rank test and the Mann-Whitney U test and the GraphPad Prism software version 5.0a (GraphPad Software, Inc., La Jolla, Ca, USA). One-way ANOVA followed by Tukey *post hoc* test was used to compare multiple data sets.

Determination of cutoff points for KCa3.1-mRNA expression was done according to the method described by Altman et al. [62]. Cutoff points were used to generate Kaplan Meier survival curves. Kaplan Meier survival curves and univariate/multivariate Cox regression were carried out using 12.1 STATA software (StataCorp, Texas, USA). Two-sided p-values < 0.05 were considered significant. Overall survival (OS) was calculated from the date of diagnosis by imaging to the date of death from any cause or last follow-up contact. Disease specific survival (DSS) was calculated from the date of diagnosis by imaging to the date of death from ccRCC or last follow-up contact. Progression free survival (PFS) was calculated from the date of diagnosis by imaging to the date of progression, relapse or death from any cause or last follow-up contact.

## Results

We examined a total of 97 ccRCC and 11 oncocytomas together with corresponding normal unaffected cortical tissue from each patient for KCa3.1 expression. QRT-PCR data was generated for 96 patients with ccRCC and for all oncocytoma patients. Clinical and demographic data are presented in Table 1. Median follow-up time for ccRCC was 29 months (range 1–106 months). The 5-year overall survival (OS) was 49%, CI [0.62–0.82] and the 5-year disease specific survival (DSS) was 61%, CI [0.72–0.90]. 34 patients (35%) died during the study period.

A total of 10 ccRCC and 11 oncocytomas were examined for KCa1.1 expression, together with corresponding normal unaffected renal cortex.

### KCa3.1 and KCa1.1-mRNA expression in tumor tissue and paired healthy kidney cortex

**Fig 1:**
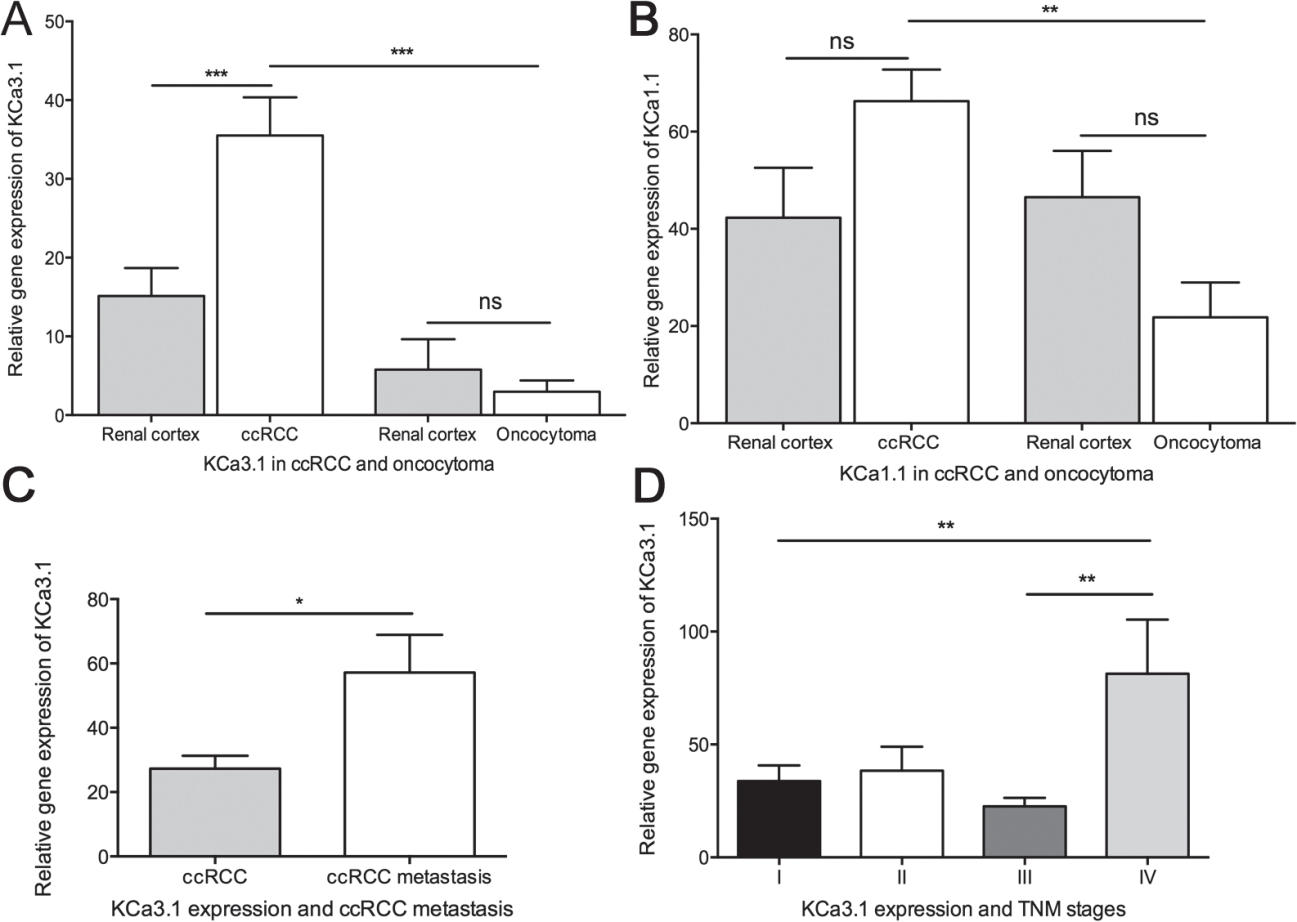
Quantitative RT-PCR analysis of KCa3.1 and KCa1.1 mRNA in oncocytoma and ccRCC together with paired normal renal cortex. Mean + SEM is shown. KCa3.1 gene expression is shown relative to reference gene expression of multiple reference genes and KCa1.1 gene expression relative to reference gene TBP. A) Comparison of KCa3.1 gene expression in tumor tissue from oncocytoma and ccRCC together with paired unaffected renal cortex, B) Comparison of KCa1.1 gene expression in tumor tissue from oncocytoma and ccRCC together with paired unaffected renal cortex C) KCa3.1 gene expression in ccRCC tumors with and without metastasis, D) Comparison of KCa3.1 gene expression in different TNM stages of ccRCC. *p < 0.05, **p< 0.01 ***p < 0.001. ns = non-significant.

We examined the difference of gene expression levels of KCa3.1 and KCa1.1 between tumor tissue and paired unaffected cortex samples from ccRCC patients and oncocytoma patients (Fig 1A and 1B). For KCa3.1, we found a significant, 2-fold higher mRNA-expression in the ccRCC tissue compared to the respective healthy cortical tissue. We did not find a significant difference between oncocytoma tissue and paired unaffected cortical tissue. A comparison of gene expression levels between tumor tissues from ccRCC and oncocytoma revealed a significant, 12-fold higher KCa3.1-mRNA expression in ccRCC, p < 0.0001 (Fig 1A). Also expression of KCa1.1 was significantly 3-fold higher in ccRCC than in oncocytoma (Fig 1B).

Regarding metastasis and tumor stage, we observed a significant, 2-fold higher mRNA level of KCa3.1 in the ccRCC tissue from patients with metastasis compared to ccRCC patients without metastasis (Fig 1C). Furthermore, mRNA expression of KCa3.1 was associated with a high TNM-grade IV (Fig 1D).

### Survival models

**Fig 2:**
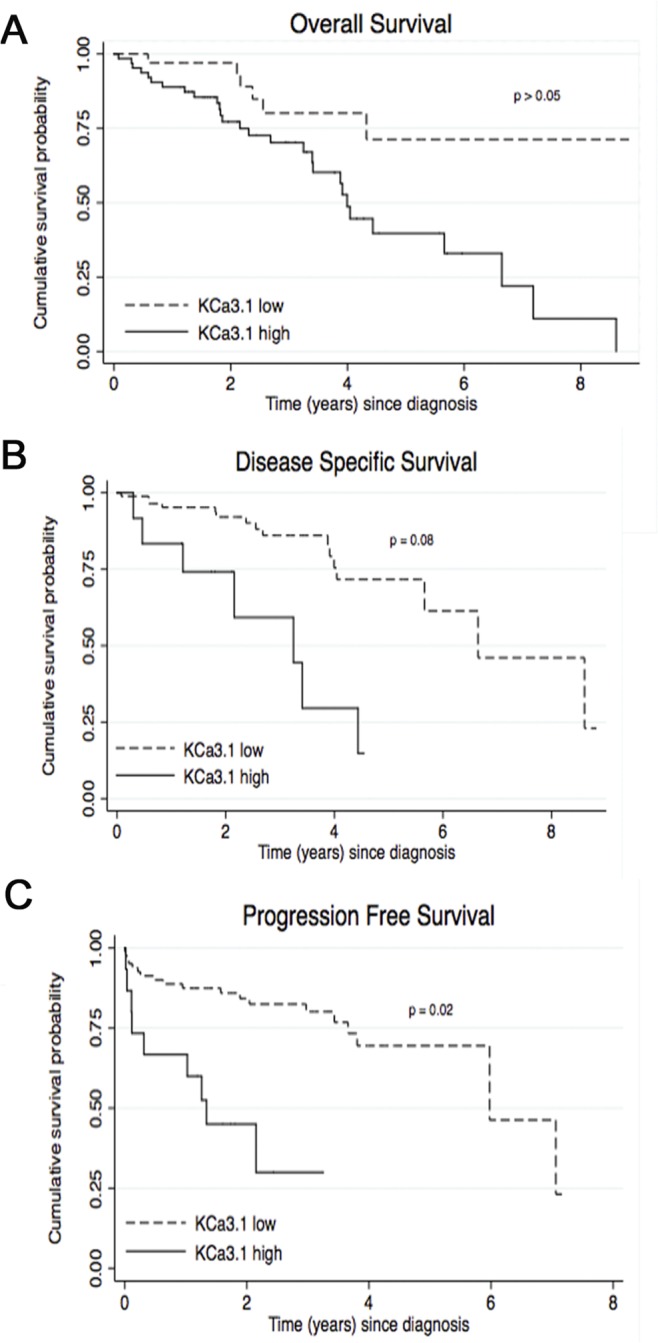
KCa3.1-mRNA expression levels are a significant prognostic indicator of Progression Free Survival in ccRCC. A-C: Kaplan Meier survival analysis indicates that patients with high KCa3.1-mRNA expression have a significantly shorter Progression Free Survival (p = 0.02). Moreover, we found a trend towards a significantly shorter Disease Specific Survival for patients with a high KCa3.1 mRNA expression (p = 0.08).

Significant cutoff points—calculated according to Altman et al. [62]—were found for KCa3.1-mRNA expression in ccRCC patients. These cutoff points were 13.6, 71.7, and 62.4 for OS, DSS, and PFS, respectively, and were used to generate Kaplan Meier curves for all three endpoints (OS, DSS, and PFS) with corrected p-values (Fig 2A–2C). OS was not significantly reduced in the subgroup with high KCa3.1-mRNA expression (Fig 2A). DSS trended towards reduction in the subgroup of high-KCa3.1-expressing tumors (p = 0.08 vs. low-KCa3.1-expressing tumors; Fig 2B). As shown in Fig 2C, PFS was significantly longer for those patients with a low KCa3.1 mRNA-expression (p = 0.02). The patients exhibiting high KCa3.1-mRNA expression developed metastasis or disease relapse after 9.4 months (± 2.4), while the patients with a low KCa3.1-mRNA expression developed metastasis after 23.9 months (± 2.7). Moreover, 60% of the patients with high KCa3.1-mRNA expression developed metastasis during follow-up time, while only 23.5% did so in the low KCa3.1-mRNA expression group.

Similarly, in the multivariate analysis (Table 3) the Cox proportional hazards model showed that high KCa3.1-mRNA expression was a predictor of early metastasis from ccRCC with a Hazard Ratio of 3.37 (p = 0.012). As expected, TNM stage and treatment with adjuvant therapy were also predictors for metastasis.

**Table 3:** Univariate and multivariate cox regression.

**ccRCC**	**Univariate Cox regression(OS) (n = 96), No of failures: 34**		**Univariate Cox regression(DSS) (n = 96), No of failures: 23**		**Univariate Cox regression (PFS) (n = 96), No of failures: 28**	
Variables	P	HR (95% CI)	P	HR (95% CI)	P	HR (95% CI)
**Sex**						
Male		1.00		1.00		1.00
Female	NS	0.8 (0.39-1.68)	NS	1.10 (.47-2.60)	NS	1.94 (.89-4.24)
**Age, yrs**						
< 64		1.00		1.00		1.00
64	0.009**	2.62 (1.28-5.38)	NS	2.19 (.92-5.19)	NS	1.30 (.62-2.74)
**TNM**						
I+II		1.00		1.00		1.00
III+IV	0.010*	2.51 (1.25-5.07)	0.007**	3.49 (1.42-8.60)	0.000***	4.16 (1.87-9.23)
**Fuhrman**						
G1+G2		1.00		1.00		1.00
G3+G4	NS	1.2 (0.6-2.4)	0.017*	3.40 (1.25-9.28)	0.000***	5.01(2.02-12.41)
**Metastasis**						
No		1.00		1.00		-
Yes	0.006**	2.75	0.000***	8.45 (2.82-25.30)	-	-
**Tumor size**						
< 7 cm		1.00		1.00		1.00
7 cm	NS	0.76 (0.37-1.52)	NS	1.29 (.54-3.06)	0.013*	2.88(1.25-6.66)
**Adj therapy**						
0		1.00		1.00		1.00
1	NS	1.10 (0.52-2.34)	NS	2.27 (.96-5.35)	0.000***	6.70(3.06-14.68)
**ccRCC (n = 97)**	**Multivariate cox regression (OS) (n = 96), No of failures: 34**		**Multivariate cox regression (DSS) (n = 96), No of failures: 23**		**Multivariate cox regression (PFS) (n = 96), No of failures: 28**	
Variables	P	HR (95% CI)	P	HR (95% CI)	P	HR (95% CI)
**TNM**						
I + II		1.00		1.00		1.00
III + IV	NS	1.30 (.56-3.01)	0.037*	2.66 (1.06-6.68)	0.006**	3.49 (1.42-8.54)
**Age, yrs**						
< 64		1.00		-		-
64	0.025*	2.58 (1.13-5.89)	-	-	-	-
**Metastasis**						
No		1.00		1.00		-
Yes	0.02*	2.55 (1.16-5.63)	0.006**	5.10 (1.61-16.12)	-	-
**Fuhrman**						
G1 + G2		-		1.00		1.00
G3 + G4	-	-	NS	1.59 (.53-4.75)	NS	2.56 (0.93-6.99)
**Tumor size**						
< 7 cm	-			-		1.00
> 7 cm	-	-	-	-	NS	0.61 (0.22-1.71)
**Adjuvant therapy**						
No	-			-		1.00
Yes	-	-	-	-	0.001**	4.68 (1.88-11.67)
**KCNN4**						
Low		1.00		1.00		1.00
High	NS	2.22(.83-5.93)	NS	1.96 (.73-5.30)	0.012*	3.37 (1.30-8.72)

* p-value < 0.05

**p-value < 0.01

*** p-value < 0.001

NS: non-significant

### Localization of KCa3.1 and KCa1.1 in tumor tissues

**Fig 3:**
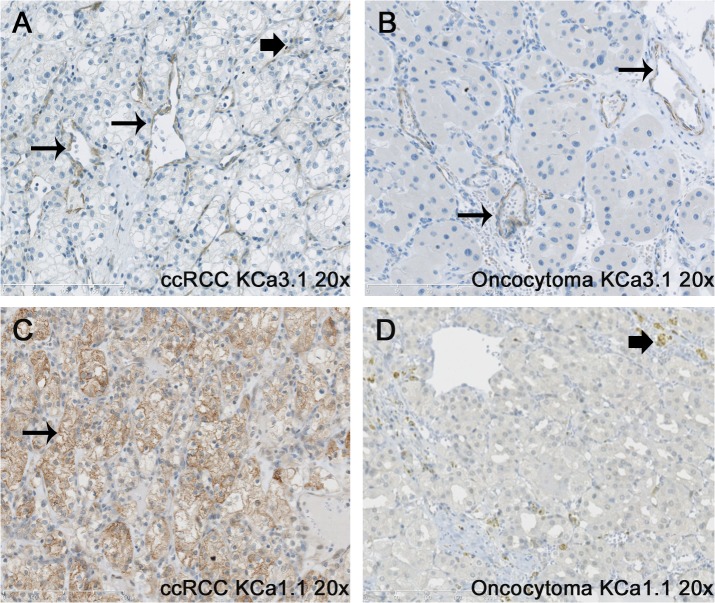
Immunohistochemical staining for KCa3.1 in ccRCC and oncocytoma. Immunohistochemical staining for KCa3.1 in ccRCC (A) and oncocytoma (B) shows strong staining of tumor vessels (long arrows) in both tumor subtypes. In ccRCC, a few tumor cells or possibly stroma cells show some KCa3.1 expression (“block” arrow). Immunohistochemical staining for KCa1.1 in ccRCC (C) and oncocytoma (D) shows staining of the cell membrane of the tumor clear cells (long arrow), whereas no staining of the tumor cells was observed in the oncocytoma. “Block” arrow indicates staining of immune cells. Original magnification, x200.

Immunohistochemistry revealed that KCa3.1 protein localized predominantly to tumor vessels of ccRCC tissue (Fig 3A). In addition, we found KCa3.1 protein in a few scattered cells within the tumor tissue. However, most of the ccRCC tumor cells did not show staining, suggesting that only a subgroup of ccRCC cells and, possibly, single stroma cells and infiltrating immune cells were KCa3.1-positive and gave rise to qRT-PCR signals together with KCa3.1-positive tumor vessels. In oncocytoma cells, we were not able to demonstrate KCa3.1-protein staining but detected, similarly to ccRCC, KCa3.1 protein in tumor vessels (Fig 3B).

The ccRCC specimens showed strong immunoreactivity to KCa1.1 at the level of the cell membrane whereas oncocytoma specimens showed either none or weak, diffuse (unspecific) immunoreactivity of the entire cytoplasm (Fig 3C and 3D). Immunoreactivity at the level of the cell membrane was not evident in oncocytoma specimens.

**Fig 4:**
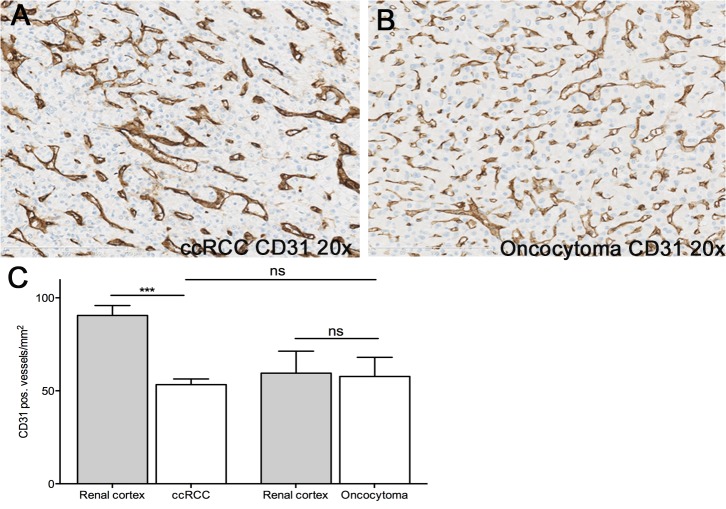
Immunohistochemical staining for CD31 in ccRCC and oncocytoma. Immunohistochemical staining for CD31 in ccRCC (A) and oncocytoma (B) showed no difference in microvessel density between the two tumor subtypes (C). Note that the unaffected cortex from ccRCC patients contained more CD31-positive blood vessels while the number of vessels was alike in oncocytoma and paired cortex. Data are given as mean ± SEM. Number of patients: n = 7; *** p<0.001 vs. ccRCC, One-way ANOVA followed by Tukey post hoc test.

The degree of tumor vascularization as determined by endothelium-selective CD31-staining was not significantly different between oncocytoma and ccRCC (Fig 4A–4C).

**Fig 5:**
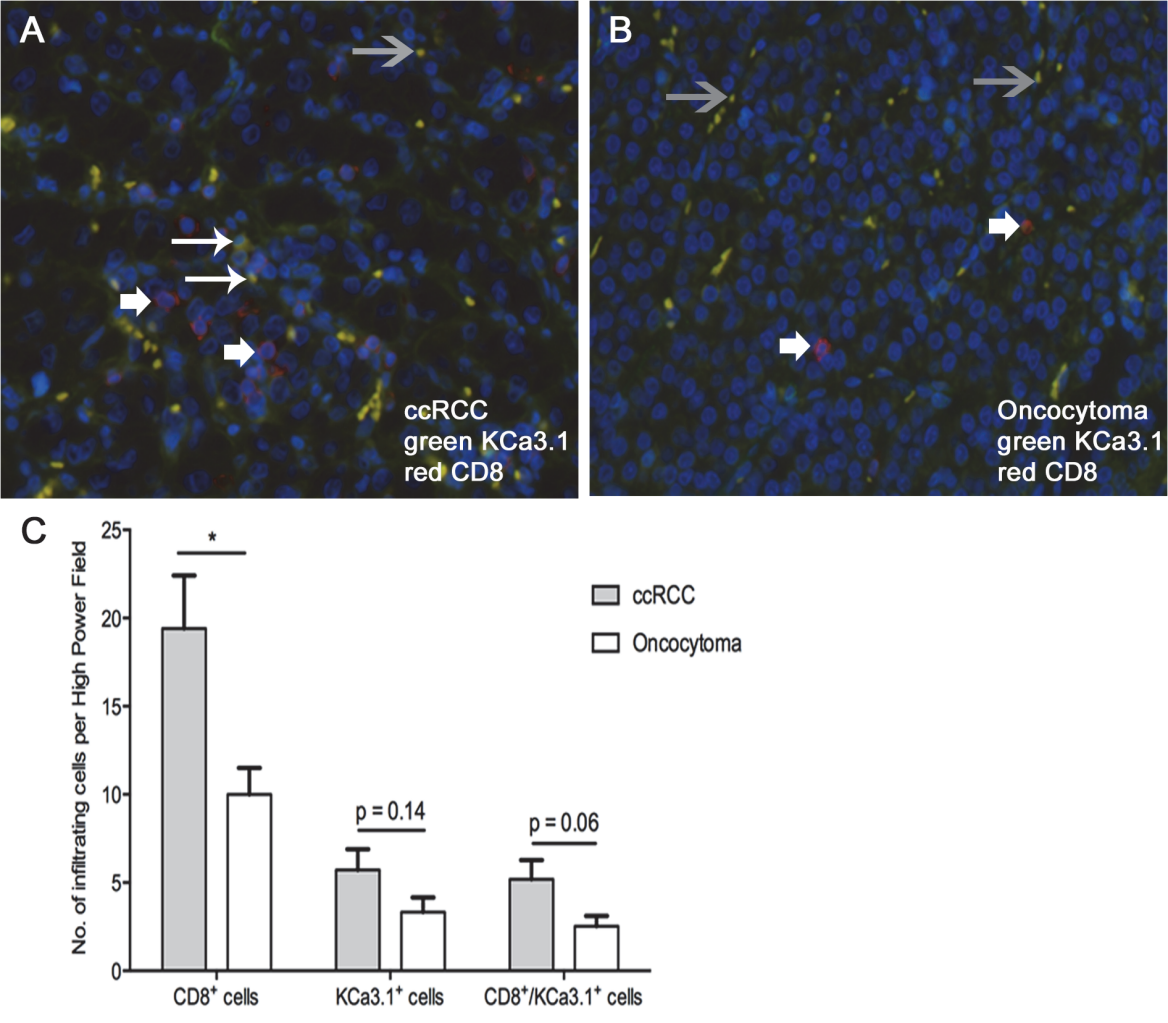
Immunofluorescent staining for CD8 and KCa3.1 in ccRCC and oncocytoma. Immunofluorescence of CD8 (red) and KCa3.1 (green) in ccRCC (A) and oncocytoma (B). Large arrows indicate T cells positive for CD8 and KCa3.1. Small arrows indicate T cells positive for CD8 but KCa3.1-negative. Grey arrows indicate erythrocytes within tumor vessels that exhibited staining for KCa3.1 and CD8 that could be, however, auto-fluorescence. (C) Quantification of CD8-positive T cells, KCa3.1-positive CD8 T cells, and other cells in ccRCC and oncocytoma tumor tissues. Data are given as mean ± SEM. Number of tumors: n = 7, each; * p<0.05 ccRCC vs. oncocytoma, One-way ANOVA followed by Tukey *post hoc* test.

KCa3.1 is expressed in cytotoxic CD8 T cells, in which the channels contribute to calcium-dependent T cell activation [63] and possibly tumor immunogenicity. When we stained infiltrated CD8 T cells in ccRCC and oncocytoma tissue we found a small number of CD8 T cells in both tumors (Fig 5A). Nonetheless, the number of CD8 T cells in ccRCC was twofold higher than in oncocytoma (Fig 5B). The co-staining revealed that KCa3.1-protein was present in CD8 T cell infiltrates of ccRCC, albeit the intensity of the signal varied substantially, and KCa3.1-protein was found in one third of the CD8 cells (Fig 5C). Overall, fluorescence signals for KCa3.1-protein appeared weaker when compared to the stronger signals coming from tumor vessels and erythrocytes passing through them (Fig 5A and 5B), and perhaps similar to that in the tumor cells themselves (Fig 3A). Nonetheless, this still suggests a contribution of KCa3.1 protein in CD8 T cells to the total KCa3.1 protein content of ccRCC.

**Fig 6:**
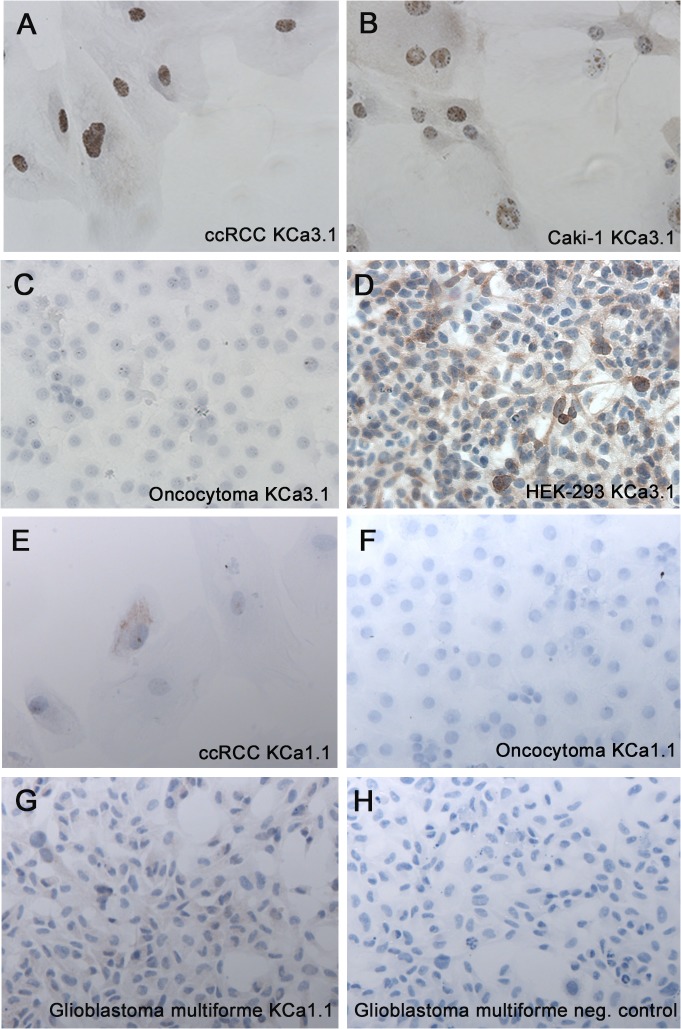
Immunocytochemical staining for KCa3.1 in a ccRCC cell line. Immunohistochemical staining for KCa3.1 in a primary ccRCC cell line showed relatively weak and heterogeneous membrane staining and an intense staining of presumably the endoplasmic reticulum around nuclei (A). Similar pattern of KCa3.1-staining was seen in Caki-1 cells (B), while primary oncocytoma cells lacked KCa3.1-stain (C). KCa3.1-transfected HEK cells served as a positive control (D). Immunohistochemical staining for KCa1.1 in primary ccRCC showed a weak staining of the membrane (E), whereas no staining was observed in the primary oncocytoma (F). A glioblastoma cell line (U251 MG) served as positive control for KCa1.1 (G-H). Original magnification, 200x.

Next, we stained the primary ccRCC and oncocytoma cell lines, as well as the ccRCC cell line, Caki-1. Again a subset of ccRCC cells showed positive KCa3.1 staining, while oncocytoma cells did not (Fig 6A–6C). Positive tumor cells were counted for each cell line and compared by a Fisher’s exact-test, which revealed that 31 out of 32 ccRCC tumor cells were positive for KCa3.1 while only 2 out of 108 oncocytoma cells were positive (p<0.0001).

Interestingly, the KCa3.1 staining of ccRCC cells was prominent around the nuclei of the ccRCC (presumably the site of protein synthesis) and relatively weak at the cell membrane level, which of note is to be expected for this type of clear cell morphology and the relatively low protein-expression rate of many ion channels in general. KCa3.1-protein was also detectable in Caki-1 cells with a similar cellular pattern, although the staining was in general more intense in these Caki-1 cells when compared to primary ccRCC cells (Fig 6B).

Staining of the primary ccRCC and oncocytoma cell lines for KCa1.1 revealed the presence of the protein in ccRCC cells, whereas no staining was seen in the oncocytoma cell line (Fig 6E and 6F).

### Electrophysiological characterization of KCa3.1 and KCa1.1 in ccRCC

**Fig 7:**
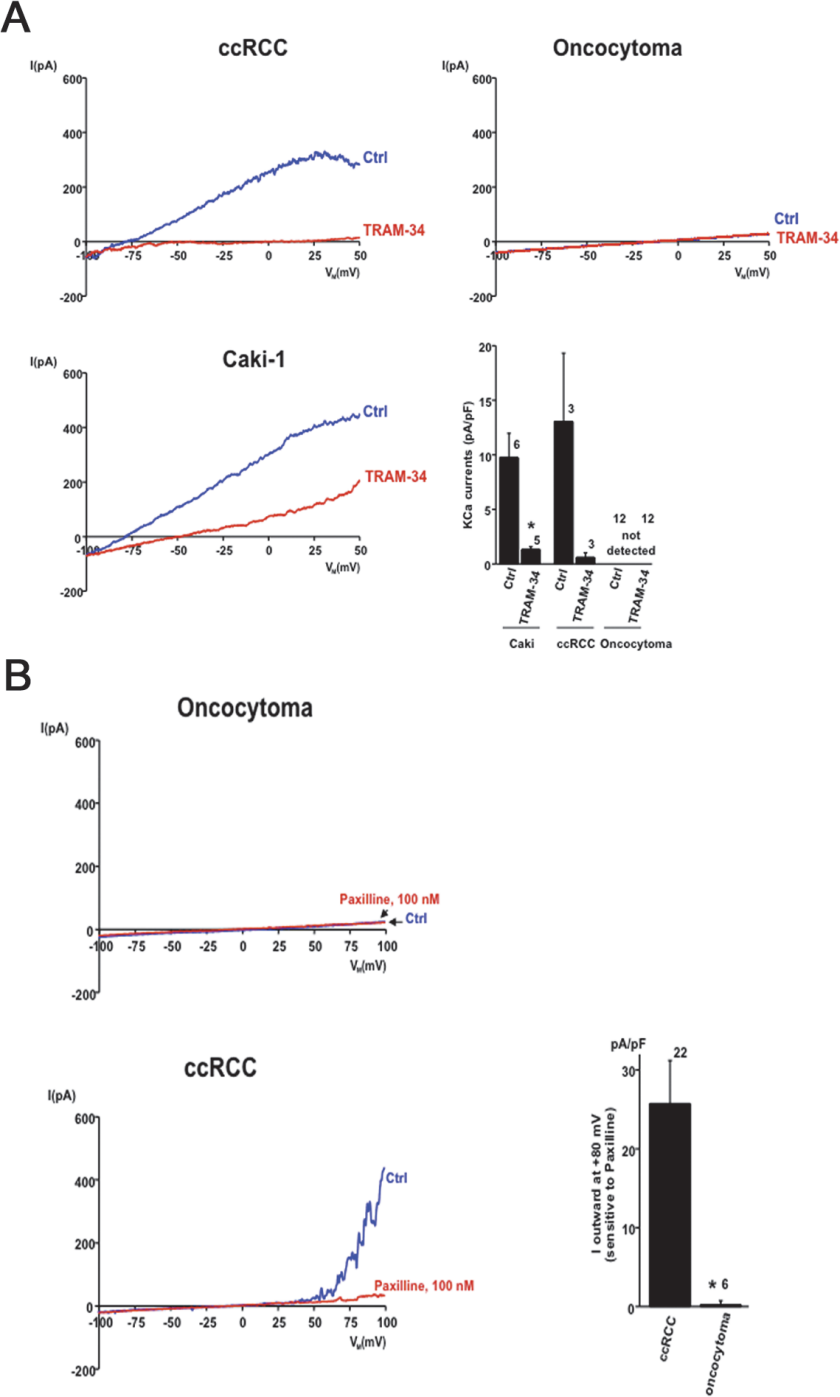
Positive KCa3.1 currents detected in ccRCC by patchclamping. (A) Electrophysiological characterization of KCa3.1 channels in ccRCC. In primary ccRCC, KCa3.1 whole-cell currents exhibited typical KCa3.1 features such as inward rectification at positive voltage and complete inhibition by the selective KCa3.1 blocker, TRAM-34. KCa3.1 currents were not detected in any of the oncocytoma cells. Caki cells displayed consistently TRAM-34-sensitive KCa3.1 currents. The graph shows summary data of KCa3.1 current densities and blockade of KCa3.1 by TRAM-34. (B) Electrophysiological characterization of KCa1.1 channel in ccRCC. In primary ccRCC, we saw a KCa1.1-typical voltage-dependent I/U relationship with large current amplitudes at positive membrane potentials. KCa1.1 currents were large in ccRCC, sensitive to Paxilline, and virtually undetectable in oncocytoma. Data are mean ± SEM. Number in graph indicate the number of repetitions; *p< 0.05.

We performed whole-cell patch-clamp experiments on primary ccRCC, oncocytoma, and Caki cells (Caki-1 and Caki-2) to demonstrate KCa3.1 and KCa1.1 currents and thus membrane-expression of the channels. The data and exemplary current traces are shown in Fig 7A and 7B. In primary ccRCC, we found modest membrane expression of KCa3.1, since we detected Ca^2+^-activated K^+^ currents with a KCa3.1-typical electrophysiological fingerprint in 3 out of 27 cells (11%). The currents showed inactivation of outward currents at positive voltage (inward rectification) and were completely abolished by the selective KCa3.1 blocker, TRAM-34 [64] (Fig 7A). Such KCa3.1 currents were not seen in a total of 12 oncocytoma cells (Fig 7A). Caki cells displayed such KCa3.1 currents consistently and TRAM-34 blocked these currents entirely (Fig 7A).

We next examined functional expression of KCa1.1 channels in ccRCC, Caki-cells and oncocytoma. KCa1.1 channels in the primary ccRCC and in Caki cells displayed the KCa1.1-typical voltage-dependent I/U relationship with large current amplitudes only at positive membrane potentials beyond 50 mV (Fig 7B). Such KCa1.1 currents were virtually undetectable in oncocytoma (p< 0.05) (Fig 7B). Paxilline, an established small molecule inhibitor of the KCa1.1 channel [65] inhibited the K-outward currents in ccRCC (Fig 7B). In contrast 1 μM of BMS 191011, a potent KCa1.1 channel opener [66], potentiated these currents (data not shown).

### Pharmacological inhibition of KCa-channels did not considerably affect Caki cell proliferation and migration

KCa3.1 and KCa1.1 have been suggested to contribute to mitogenesis [12,34,44,45]. In light of our observations that TRAM-34 and Paxilline inhibited KCa3.1 and KCa1.1 currents, respectively, we tested whether pharmacological inhibition of KCa1.1 and KCa3.1 channels decreases ccRCC cell proliferation in vitro (S2 Fig). Inhibition of KCa3.1 by TRAM-34 reduced Caki cell proliferation to a minor degree (≈10%). However, Paxilline and the combination of Paxilline and TRAM-34 did not significantly inhibit Caki cell proliferation.

We also performed a cell scratch assay to study whether the channels contribute to mechanisms of cell migration. However, neither Paxilline, another KCa3.1-blocker, RA-2 [61], nor a combination of the two blockers appreciably affected closing of the scratch wound (S3 Fig).

## Discussion

In the present study we identified the KCa3.1 channel as a molecular marker of ccRCC with significant prognostic value for PFS in ccRCC patients. The following experimental evidence fostered this view: 1) KCa3.1-mRNA levels were 12-fold higher in ccRCC compared to the benign tumor, oncocytoma, and 2-fold higher than in healthy cortical tissue. Furthermore, a high expression of KCa3.1-mRNA levels correlated with TNM stage IV. 2) High mRNA-expression levels in ccRCC gave a poorer prognosis with lower PFS. 3) Our cell biological studies showed that KCa3.1 is located in a small subset of ccRCC cells and possibly stroma cells within the tumor and in tumor vessels, while benign oncocytoma cells were devoid of KCa3.1 channels. 4) The KCa3.1-blocker, TRAM-34, produced a small but significant reduction of Caki-1 cell proliferation *in vitro*. Together, these results identified KCa3.1 as a new molecular marker of disease progression and survival in ccRCC.

Differential regulation of ion channels is considered a feature of a variety of tumors and has been suggested to be involved in cancer progression and metastasis [67–70]. Among the different types of channels, in particular, KCa3.1 but also the related KCa2.3 channel [71,72] of the same gene family, was found to be up-regulated in some but not all cancers and especially in those KCa3.1-expressing tumor cells showing high activity of MAP kinase cascades and AP-1 activity [17]. However, most of the evidence derived from in-vitro experimentation that is sensitive per se to cell culture artifacts (exposure to mitogens in cell culture media and supplements) and rather little was known about KCa3.1 protein expression in the original tumor tissue, which was mainly due to ineffective immunohistochemical approaches using antibodies of uncertain specificity [57]. Moreover, from past experiments on tumor samples, it remained unclear whether high KCa3.1-mRNA expression studies or western blotting detected KCa3.1 channels in tumor cells, in stroma or in the tumor vasculature. Indeed, mitogen-induced up-regulation of KCa3.1 has been shown to occur particularly in proliferating human endothelium [20], smooth muscle [26], and connective tissue (fibroblasts) [73]. Moreover, KCa3.1 signals might derive from infiltrating immune cells [74]. Recent molecular and immunohistochemical evidence from our and other groups has shown that KCa3.1 is up-regulated at the mRNA transcription level as well as at the protein level in glioblastoma [12,34,57]. Indeed, KCa3.1up-regulation was found to be of prognostic value since expression levels had an impact on invasiveness of glioblastoma cells [12]. Besides the well-established KCa3.1-up-regulation in glioblastoma, there was good evidence for KCa3.1 up-regulation in mammary carcinoma [13,16] and colonic adenocarcinoma [21], although the diagnostic or prognostic value of this remained largely obscure. Our present study on KCa3.1 in ccRCC added to the current knowledge because we showed that KCa3.1 was strongly up-regulated in a large number (n = 96) of malignant ccRCC compared to benign oncocytoma (n = 11). IHC revealed further that KCa3.1 protein expression was restricted to a small subset of ccRCC cells. Importantly, this up-regulation was not just an observational finding at the mRNA transcription/translation level, since we demonstrated KCa3.1-membrane expression by patch-clamp in the ccRCC cells, although at low frequency. In this respect, KCa3.1 expression in ccRCC differed substantially from that in Caki cells (this study) and glioblastoma, in which virtually all tumor cells are KCa3.1-positive [57].

Strikingly, the endothelium of the ccRCC vasculature showed very high KCa3.1-protein expression, which is in line with the constitutive expression of KCa3.1 in the endothelium of blood vessels and, in addition, with up-regulation of KCa3.1-mRNA expression and functional KCa3.1-proteins (currents) in human endothelial cells after mitogenic stimulation as shown previously by us [20]. Notably, the endothelial channel has been shown previously to be associated with colonic adenocarcinoma as concluded from the higher mRNA and membrane expression of KCa3.1 in tumor-near mesenteric arteries from adenocarcinoma patients [21]. It is likely that the finding of higher KCa3.1-mRNA-expression in ccRCC compared to oncocytoma could be explained at least in part by higher KCa3.1-mRNA expression in the tumor vessels. However, ccRCC samples did not harbor more blood vessels than oncocytoma samples. Still, it is possible that a higher KCa3.1-mRNA-expression is present in ccRCC tumor vessels, although the IHC staining as non-quantitative measurement of KCa3.1 protein− appeared alike in the two tumors.

The degree of CD8 T cell infiltration, as a possible source of KCa3.1-mRNA and perhaps relevant for cancer immunology, was significantly higher in ccRCC than in oncocytoma. However, amounts of KCa3.1 protein were variable in these cells as concluded from co-staining experiments. Still, this raised the possibility that expression of KCa3.1 in cytotoxic CD8 T cells contributed to the higher KCa3.1–mRNA expression found in the total tumor tissue. It also raised the intriguing possibility that immune cell KCa3.1 channels could add to tumor immunogenicity. However, whether or not KCa3.1-expressing CD8 T cells contribute positively or negatively to progression of ccRCC cannot be answered by the present study.

With respect to the prognostic value of KCa3.1-mRNA expression, we demonstrated that ccRCC patients with high KCa3.1-mRNA expression levels above cutoff showed very low PFS and high rates of metastasis, suggesting that the KCa3.1 was disadvantageous in this subgroup. This view is in line with the channel’s role in driving tumor cell and tumor invasiveness, as outlined above, as well as in endothelial cell proliferation, as concluded from pharmacological in-vitro and in-vivo studies testing anti-proliferative actions of KCa3.1 blockers like TRAM-34 on endothelial cell proliferation and artificial matrigel-vascularization [20]. Accordingly, in the present study, TRAM-34 had significant proliferation-blocking efficacy on Caki-1 cells in vitro, although the effect was rather small (≈-10%) under these in-vitro conditions. Still we speculate that pharmacological blockade might have some beneficial therapeutic effect in patients, as suggested for glioblastoma previously, but more likely as adjuvant and possibly metastasis- and tumor angiogenesis-impairing treatment in conjunction with standard chemotherapy and surgery.

In addition to KCa3.1, we identified also the distantly related, voltage- and Ca^2+^-activated KCa1.1 channel as a differentially expressed channel in the two tumor types (3-fold higher mRNA expression and presence of functional KCa1.1 currents in ccRCC but not in oncocytoma) and between healthy and tumor tissue of the same kidney. The latter observation is in line with the previously reported up-regulation of KCa1.1 gene expression in ccRCC [75]. This channel is known to contribute to physiological K^+^ secretion in the renal tubular system and collecting ducts[42,43].

Since this channel is apparently lost in oncocytoma originating from the distal tubule, it is tempting to speculate that conserved expression of this channel in ccRCC adds to oncogenesis and/or progression. Still, it may serve as marker of ccRCC, in this regard similar to KCa3.1, although up-regulation of KCa1.1 gene expression was less pronounced in ccRCC. Again similar to KCa3.1, the channel did not critically contribute to proliferation/migration of Caki-1 cells as concluded from the lack of effect of pharmacological blockade by Paxilline in our cell proliferation and migration assays. Still, our study provided the first evidence that KCa3.1 and KCa1.1-channels are differentially regulated in renal cancer at the functional level as well as at the level of gene expression. Possibly future experimental intervention studies in vivo will provide insight whether the channels are of functional importance and can be exploited for alternative, adjuvant therapy.

In conclusion, we identified the KCa3.1 and KCa1.1 channels in ccRCC patients as molecular markers of ccRCC compared to benign oncocytoma. Moreover, the KCa3.1 channel was of prognostic value as high KCa3.1-mRNA-expression levels were associated with development of metastasis and a lower survival.

## Supporting Information

S1 Fig Immunohistochemical staining for KCa1.1 and KCa3.1 in normal tissue. (A) Localisation of KCa1.1 in different segments of the nephron. Particularly the proximal convoluted tubule stains strongly, but glomerular mesangial cells and the distal convoluted tubule also exhibited expression of KCa1.1. (B) Localisation of KCa3.1 in serous acini in the human parotid gland.
(DOCX)

S2 Fig Inhibition of KCa channels does not affect proliferation of Caki-1 cells. Proliferation assay. Inhibition of KCa3.1 and KCa1.1 by TRAM-34 (1μM) and Paxilline, respectively, or of both channels by a combination of the blockers did not modulate proliferation of Caki-1 cells. Data (absorbance values) were normalized to vehicle (DMSO). Number of repetitions, n = 12. *p<0.05.
(DOCX)

S3 Fig Inhibition of KCa channels does not affect migration of Caki-1 cells. Migration assay. No significant inhibition of wound closing with either Paxilline, RA-2 or a combination of both blockers. Data are given as % of remaining cell-free area. N = 2 repetitions for each condition. Data points are mean ± SEM; *p<0.05.
(DOCX)
