# Supplementary material for: High Expression of KCa3.1 in Patients with Clear Cell Renal Carcinoma Predicts High Metastatic Risk and Poor Survival
Source: PLoS One. 2015 Apr 7;10(4):e0122992. doi: 10.1371/journal.pone.0122992 (PMC4388734; doi:10.1371/journal.pone.0122992)
Supplement: S1 Fig — (A) Localisation of KCa1.1 in different segments of the nephron. Particularly the proximal convoluted tubule stains strongly, but glomerular mesangial cells and the distal convoluted tubule also exhibited expression of KCa1.1. (B) Localisation of KCa3.1 in serous acini in the human parotid gland. (DOCX) [file pone.0122992.s001.docx]

**S1 Fig. Immunohistochemical staining of KCa1.1 and KCa3.1 in normal tissue**


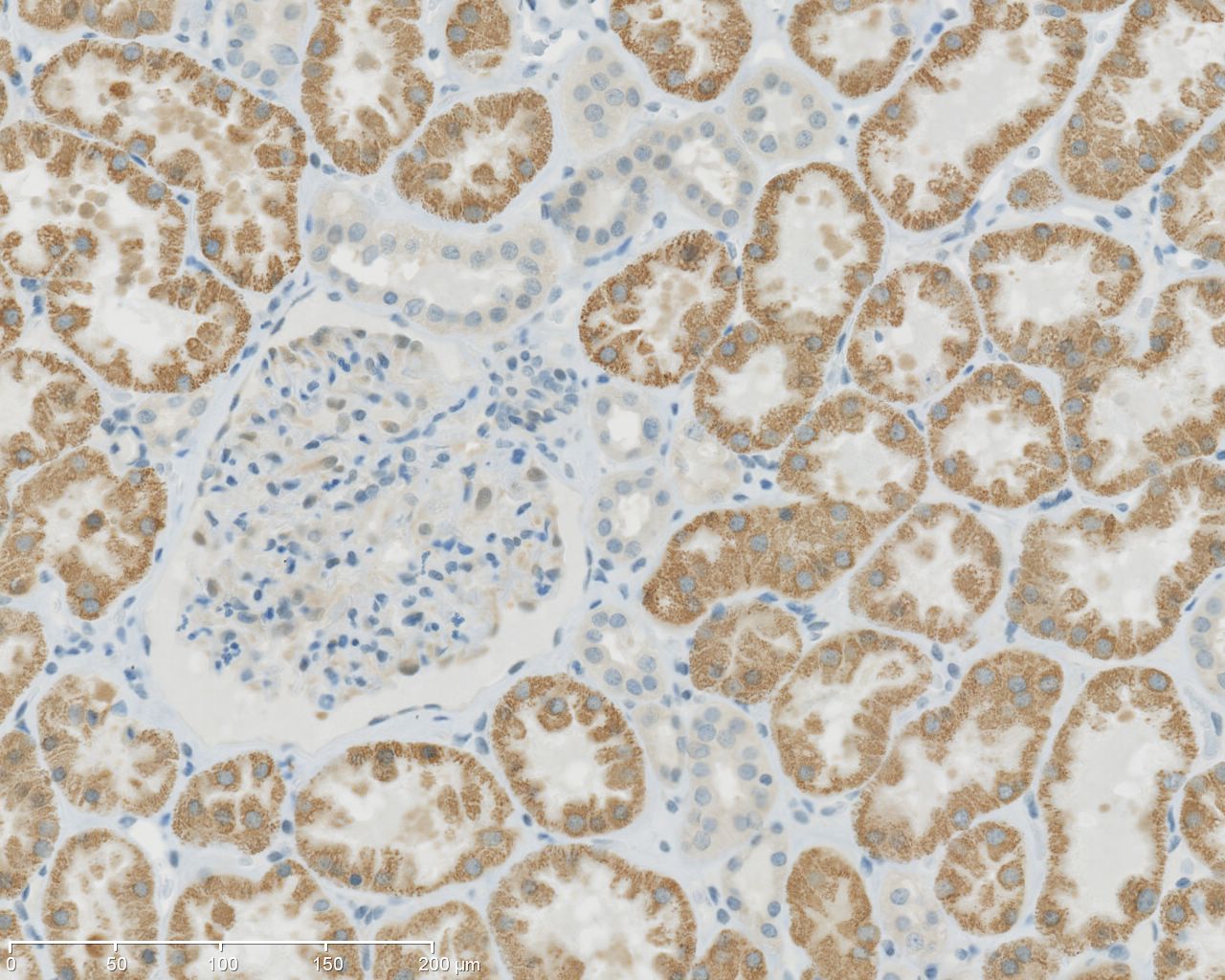
 KCa1.1 in normal kidney 20x

**A**


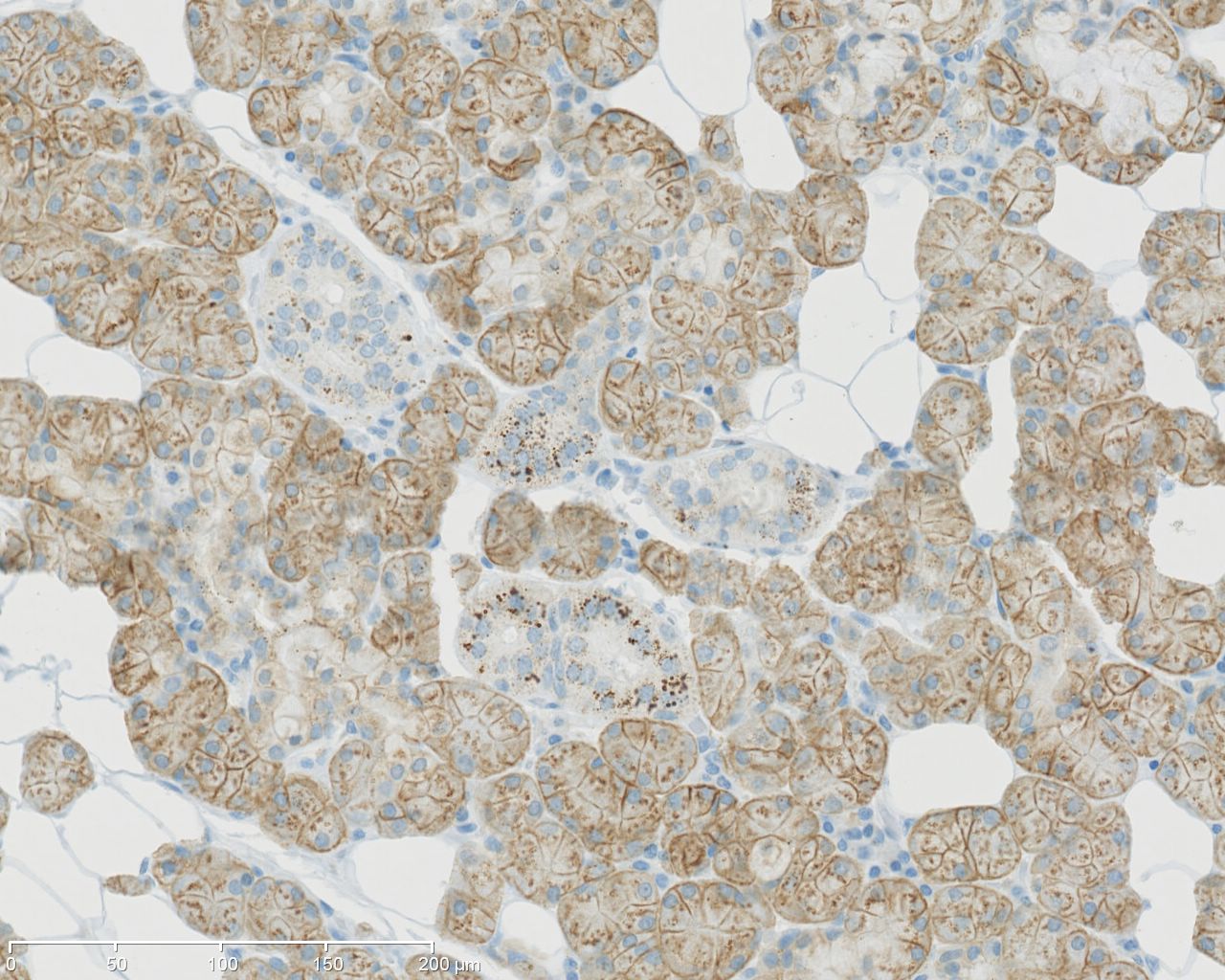
 KCa3.1 in parotid gland 20x

**B**
