# Supplementary figures and images for: High Expression of KCa3.1 in Patients with Clear Cell Renal Carcinoma Predicts High Metastatic Risk and Poor Survival
Source: PLoS One. 2015 Apr 7;10(4):e0122992. doi: 10.1371/journal.pone.0122992 (PMC4388734; doi:10.1371/journal.pone.0122992)

**S2 Fig. Inhibition of KCa channels does not affect proliferation of Caki-1 cells**


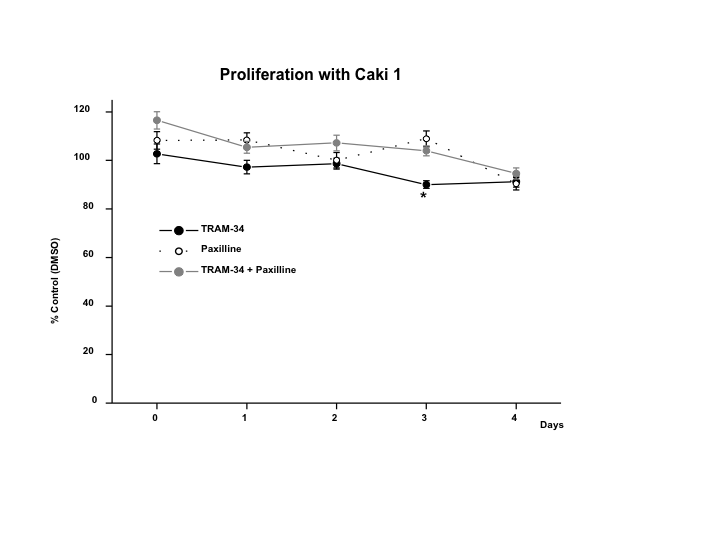

Supplement: S2 Fig — Proliferation assay. Inhibition of KCa3.1 and KCa1.1 by TRAM-34 (1μM) and Paxilline, respectively, or of both channels by a combination of the blockers did not modulate proliferation of Caki-1 cells. Data (absorbance values) were normalized to vehicle (DMSO). Number of repetitions, n = 12. *p<0.05. (DOCX) [file pone.0122992.s002.docx]

**S3 Fig. Inhibition of KCa channels does not inhibit migration of Caki-1 cells**


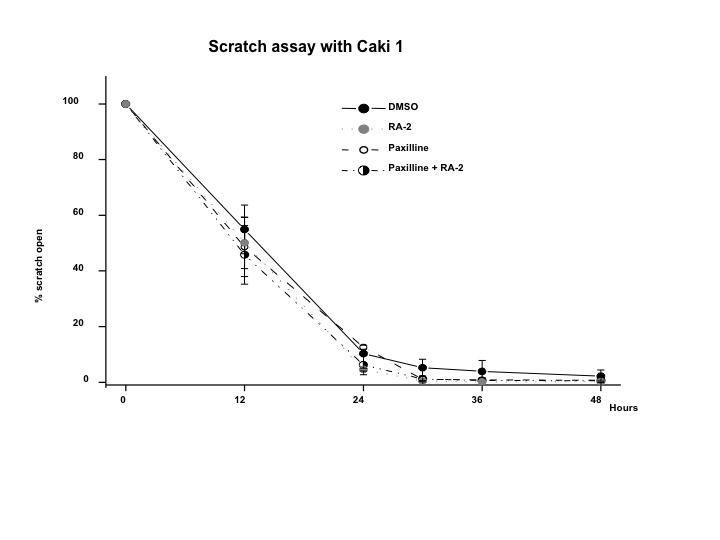

Supplement: S3 Fig — Migration assay. No significant inhibition of wound closing with either Paxilline, RA-2 or a combination of both blockers. Data are given as % of remaining cell-free area. N = 2 repetitions for each condition. Data points are mean ± SEM; *p<0.05. (DOCX) [file pone.0122992.s003.docx]
